# Taxing Meat: Taking Responsibility for One’s Contribution to Antibiotic Resistance

**DOI:** 10.1007/s10806-017-9660-0

**Published:** 2017-03-17

**Authors:** Alberto Giubilini, Patrick Birkl, Thomas Douglas, Julian Savulescu, Hannah Maslen

**Affiliations:** 1 Oxford Martin School, University of Oxford, Oxford, UK; 2 Department of Animal Bioscience, University of Guelph, Guelph, Canada; 3 Uehiro Centre for Practical Ethics, University of Oxford, Oxford, UK

**Keywords:** Antibiotic resistance, Antibiotics in animals, Taxation, Animal farming

## Abstract

Antibiotic use in animal farming is one of the main drivers of antibiotic resistance both in animals and in humans. In this paper we propose that one feasible and fair way to address this problem is to tax animal products obtained with the use of antibiotics. We argue that such tax is supported both by (a) deontological arguments, which are based on the duty individuals have to compensate society for the antibiotic resistance to which they are contributing through consumption of animal products obtained with the use of antibiotics; and (b) a cost-benefit analysis of taxing such animal products and of using revenue from the tax to fund alternatives to use of antibiotics in animal farming. Finally, we argue that such a tax would be fair because individuals who consume animal products obtained with the use of antibiotics can be held morally responsible, i.e. blameworthy, for their contribution to antibiotic resistance, in spite of the fact that each individual contribution is imperceptible.

## The Use of Antibiotics in Animals and the Problem of Antibiotic Resistance

It has been estimated that the total annual consumption of antibiotics in animal agriculture ranges from around 63,000 tonnes to around 240,000 tonnes (O’Neill 2015, p. 5), although estimates vary considerably because of lack of adequate surveillance and data collection, particularly in developing countries (Grace 2015). What we know with certainty, however, is that much antibiotic use in animals is not therapeutic; antibiotics are frequently given for prophylactic or production (i.e. growth promotion) purposes (O’Neill 2016).

As is the case with human consumption of antibiotics, the use of antibiotics in agriculture causes antibiotic resistance in bacteria (Allen 2014; Cohen and Tauxe 1986). Resistant pathogens that have been detected in livestock and food products include strains of *Campylobacter*, *Salmonella*, *Enterococcus,* and *Escherichia coli* (Graham et al. 2007, p. 80). Antibiotic resistance develops because antibiotics predominantly kill less resistant bacteria. Without competitors, more resistant bacteria proliferate and pass on their resistance-conferring genes (CDC 2015). The fact that growth-promoting antibiotics (GPAs) are normally administered in subtherapeutic doses makes them particularly conducive to selection for resistance in bacteria (Graham et al. 2007, p. 80).

Through contact with animals or consumption of animal products, resistant bacteria can be transferred to humans (CDC 2015; Liu et al. 2016). For example, farmers working with animals infected with methicillin-resistant *Staphylococcus aureus* have a higher than normal risk of being infected with these bacteria (WHO 2015, p. 3). According to the WHO’s *Global Action Plan on Antimicrobial Resistance*, “food is one of the possible vehicles for transmission of resistant bacteria from animals to human beings and human consumption of food carrying antibiotic resistant bacteria has led to acquisition of antibiotic-resistant infections” (WHO 2015, p. 3).

The risk that antibiotic use in animals will compromise antibiotic effectiveness in humans is significant. It has been estimated that, worldwide, 30% of people work in animal agriculture, and that every year farming produces 4 billion tonnes of food to feed more than 7 billion people (Grace 2015, p. 1). Considering the very widespread use of antibiotics in animal farming, the number of individuals infected with antibiotic resistant bacteria through contact with animals or food may be very high. Moreover, from 2010 to 2030, the global use of antibiotics in agriculture is predicted to increase by 67%, in part due to expanding demand for livestock products in countries such as Brazil, Russia, India, China and South Africa, where antimicrobial use in agriculture is predicted to nearly double (Van Boeckel et al. 2015).

Of particular concern is the risk of transmission to humans of bacteria that are resistant to the antibiotics that humans need the most. In the US, 62% of the antimicrobials that were sold for use in animals in 2014 are considered medically important for human health by the Food and Drug Administration (FDA 2015, p. 17). Some of the antibiotics used in animals are last-line antibiotics for humans. One example is colistin, which is our last defense against many multi-resistant bacteria, particularly bacteria which are resistant to carbapenem antibiotics (O’Neill 2015, p. 13): colistin-resistant *E. coli* has been found in more than 20% of animals in areas of China where colistin is routinely given to pigs (Liu et al. 2016).

In this paper we argue that, because antibiotic use in animals has a cost for society in terms of loss of antibiotic effectiveness, those who contribute to such use by knowingly buying animal products obtained with the use of antibiotics should be taxed at a level that at least partially (if not fully) internalizes the costs. Moreover, we argue that the proceeds from these taxes should be used to compensate those harmed by antibiotic resistance or to compensate society more generally, for example by fostering the development of practices that will diminish future antibiotic resistance. More precisely, in “The Justification for a Tax on Meat Produced with the Use of Antibiotics” section we will present two kinds of arguments for a tax on animal products obtained with the use of antibiotics. First, from a deontological perspective, if consumers are informed about the use of antibiotics in the production of the meat they buy and about the consequences of antibiotic use in terms of antibiotic resistance, they have a duty to compensate society for their contribution to the creation of antibiotic resistance. Second, from the point of view of a cost-benefit analysis, the benefits of requiring consumers to internalize the cost of antibiotic resistance, for example through taxation, seem to outweigh the costs. More in particular, internalization of the cost of antibiotic resistance could have two significant benefits: it could discourage consumption of meat produced with the assistance of antibiotics and thus diminish the social costs of resistance; and it could generate revenue that could be used to fund alternatives to antibiotics used for prophylactic, therapeutic, and growth promoting purposes. Also this support of alternative methods could be seen as a form of compensation for society-at-large because these alternatives would ensure that society, which is now harmed by the use of antibiotics in animal farming, will be better off in the long run as a result of the reduction of antibiotic resistance.^1^

Introducing a tax on agricultural products produced with the aid of antibiotics is preferable to an outright ban on antibiotic use in animal farming, because producers would probably not be able to afford the cost of transitioning immediately to an antibiotic-free system of meat production; however, revenue generated by taxation could be used to fund this transition. In “Tax to Fund Alternatives to Antibiotics Used for Prophylactic or Therapeutic Purposes” section and “Tax to Fund Alternatives to Growth Promoting Antibiotics” section we will explain how a tax could contribute to funding the transition to a system of production in which (a) prophylactic and therapeutic use of antibiotics is more responsible (“Tax to Fund Alternatives to Antibiotics Used for Prophylactic or Therapeutic Purposes” section) and (b) antibiotics are no longer used as growth promoting agents (“Tax to Fund Alternatives to Growth Promoting Antibiotics” section). Finally, in “Collective and Individual Responsibilities” section, we address a possible objection to our argument: we explain why imposing such a tax on those who consume animal products obtained with the use of antibiotics would be legitimate even if any individual contribution to antibiotic resistance through meat consumption is imperceptible, and even if the responsibility for antibiotic resistance is essentially collective, and not individual.

## The Justification for a Tax on Meat Produced with the Use of Antibiotics

In this section we are going to present a deontological reason and reasons based on a cost-benefit analysis in support of a tax on meat produced with the use of antibiotics. The deontological reason is that there is a duty to compensate society for imposing a cost on it, which translates into a duty to internalize the cost, at least as much as is possible and reasonable to do, for a negative externality produced. As for the reasons based on a cost-benefit analysis, one is that a consumption tax would probably discourage people from buying meat produced with the use of antibiotics, thus disincentivising the use of antibiotics in animal farming; a second reason is that the tax would generate revenue that could be used to fund alternatives to antibiotic use in animal farming, thus reducing the impact of antibiotic use on antibiotic resistance. Let’s consider these arguments in order.

### Deontological Reasons

Antibiotic resistance is a negative externality of antibiotic use. A negative externality can be defined as the cost of a certain practice or exchange to a third party that has not chosen to bear the cost. Antibiotic resistance is a negative externality because it reduces the availability of effective antibiotics for everyone, without their consent, thus harming society at large. We can assume that individuals have a prima facie duty not to harm others, including when the harm is “identity independent”, i.e. when *any* person living now or in the future might be harmed (Anomaly 2009, p. 430), for example any individual who will not be able to use effective antibiotics due to antibiotic resistance. Antibiotic resistance is driven, among other things, by antibiotic use and abuse in animal farming. Consequently, consumers’ choices to buy animal products obtained with the use of antibiotics, to the extent that they support the use of antibiotics in animal farming, are harming people. Those who consume such animal products are therefore violating a prima facie moral duty not to harm others. The fact that they are violating a moral duty not to harm others generates a *prima facie* moral duty for them to compensate those they are harming. A tax on consumption of non antibiotic-free meat can therefore be justified on the basis of this duty of compensation, provided the tax is used to benefit society. This is a deontological argument for a tax on meat produced with the use of antibiotics.

Now, harming others would *not* generate a corresponding duty to compensate those harmed only if those who harm others were not morally responsible, i.e. not morally blameworthy, for the harm to which they are contributing. Being morally responsible for a certain outcome presupposes being aware that one’s choices will result in that outcome, or at least being responsible for one’s lack of awareness. Most consumers of animal products are probably not aware of the use of antibiotics in animal farming and of its consequences, because labelling animal products with reference to antibiotic use is currently not mandatory in any country [although some producers have voluntarily introduced this type of labelling (O’Neill 2015, p. 26)]. Accordingly, at present, we can grant that most consumers are not fully morally responsible for the negative externalities of the current system of meat production. But this state of affairs can and should be changed.

It can be argued that consumers ought to be provided with more information about the use of antibiotics in the production of food and its possible contribution to antibiotic resistance. In fact, this may be desirable independently of the issue of consumers’ responsibility for antibiotic resistance. Generally speaking, there are reasons to provide consumers with as much information relevant to their choices as possible, for example through labelling products, so as to put consumers in the position to make better informed choices.^2^ It is worth noting that consumers seem to be more sensitive to information about antibiotic use than one might initially think. Actually, there are reasons to think that providing consumers with this type of information might even have some benefits for producers, and at the same time might lead to a decrease in antibiotic use in animal agriculture. For example, a 2012 study suggests that more than 60% of consumers in the US would be willing to pay 5 cents per pound more for antibiotic-free meat (Consumer Reports 2012, p. 8). Sales of chicken labelled “antibiotic-free” has increased by 34% in 2013 (O’Neill 2015, p. 26). One of the reasons why Sweden banned GPAs in 1986 was that farmers themselves requested so, after learning from a 1984 report that consumers’ confidence in meat safety dropped when consumers were made aware of the massive use of GPAs in animal farming (Cogliani et al. 2011)—as a result, use of antibiotics in animals in the country decreased by approximately 55% in the period 1986–1999 (Castanon 2007). Thus, there are reasons, grounded in empirical facts, to think that introducing labels referring to antibiotic use might allow many consumers to make exactly the kind of informed choice they already want to make.

More importantly for the present purposes, labels referring to both antibiotic use and its consequences in terms of antibiotic resistance would also make people more morally responsible for the consequences of their choice. It is only when people are morally responsible that they can be required to compensate society, and as we shall see in the next section, requiring individuals to compensate society for the harm they are creating could be beneficial to society, depending on how the tax is used. This is a further reason why labelling animal products with reference to antibiotic use is desirable. A simple “no antibiotic” label, or—as proposed by the UK Review on Antimicrobial Resistance (O’Neill 2015, p. 26)—a label of “responsible use of antibiotics” might not be informative enough to make consumers fully morally responsible. But, labels that in addition warn that use of antibiotics in animal farming leads to antibiotic resistance would provide consumers with all the relevant information that is necessary to make them fully morally responsible for the negative externality of a practice that they are supporting through their choice of consuming certain products: individuals would be blameworthy for failing to read the label or for disregarding the information provided. Accordingly, once this information is made available to them, there would be strong reasons to request these consumers to compensate society for the acceleration of antibiotic resistance that they are knowingly causing, for example by paying a tax on consumption of animal products obtained with the use of antibiotics. It seems reasonable to claim that the fact that certain people are *knowingly* imposing an *avoidable* cost on society-at-large generates a *prima facie* moral obligation to compensate society-at-large for such cost, because where individuals know about the consequences of their choices and have the option to buy alternative products (i.e. either antibiotic-free meat or vegetarian options) they can choose whether or not to contribute to the social cost. Thus, taxing consumers in order to force them to compensate society for the cost they are *knowingly* imposing on it seems to be justified on the basis of a moral duty of compensation that individuals anyway have. In the next section we will see how a possible way of compensating society consists in using revenue from the tax to fund alternatives to antibiotic use in animal farming.

But to what extent should individuals compensate others for the cost they are imposing on them? It seems fair to claim that individuals should at least partially *internalize* the cost of antibiotic resistance. Internalization is typically achieved by “taxing negative externalities (…) at a rate that would offset the social cost of the activities that generate the externalities, and then (ideally) using the revenues from the tax to fund socially useful projects” (Anomaly 2009, p. 433). A tax intended to internalize negative externalities is known as “Pigovian tax” (named after the 20th century economist Arthur Pigou). Admittedly, it might not be possible to fully internalize the cost of antibiotic resistance, i.e. to make sure that purchases of animal products are subject to a fee equivalent in value to the social cost in terms of increased antibiotic resistance. Internalization might only be partial, i.e. the price faced by consumers might still be too low to fully reflect the social costs they impose. For instance, partial internalization might be imposed in cases in which a full internalisation would pose an unreasonably high cost on consumers. Whether full internalization is feasible also depends on whether the cost of antibiotic resistance caused by use of antibiotics in animal farming can be quantified. But quantifying such cost would be very difficult, not least because antibiotic resistance is a global problem, not confined to single states (Rudholm 2002) or to individual antibiotic consumption. In any case, whether or not it would represent a full or only a partial internalization, there seems to be a strong deontological justification in support of taxing individuals for producing negative externalities: those who are morally responsible for a negative externality have a prima facie moral obligation to bear the cost for it, or at least to bear as much of the cost as is possible or as is reasonable.

Thus, the introduction of a Pigovian tax on consumption of animal products seems to be justified from a deontological perspective. Indeed, the compensation of those affected by negative externalities and the internalization of such externalities of production and consumption of certain goods are commonly regarded as legitimate objectives of taxation, and of consumption taxes in particular (Halliday 2013, p. 1119). As Daniel Halliday put it, a consumption tax is justified in that it “either forces the consumer to internalise the costs of their activity or to generate revenues that can be used to compensate those affected by the externality” (Halliday 2013, p. 1119). For example, one of the reasons why alcohol and tobacco consumption is usually taxed is that in this way the social costs, e.g. in terms of health expenditure, of drinking and smoking are internalized by drinkers and smokers. Granted, as said above, quantifying the social cost of antibiotic resistance might not be as easy as quantifying the social cost of alcohol or tobacco consumption. But in any case, there are strong reasons for requiring those who knowingly cause antibiotic resistance to at least compensate society for the harm, and to contribute as much as possible towards the internalization of the social cost.

It is worth noting that, from a deontological perspective, taxation is justified regardless of whether antibiotics are used for therapeutic, for prophylactic, or for growth purposes. A social cost is produced by antibiotic use regardless of the purpose of this use, and the cost is determined in part by consumers’ choice to buy certain animal products when alternatives are available. As put by the Review on Antimicrobial Resistance, “every time a farmer uses antimicrobials a cost is created for the whole society, regardless of whether the use of an antimicrobial is justified or not” (O’Neill 2015, p. 29). It is true that when they are used for therapeutic purposes antibiotics are necessary to preserve the health of animals. But such necessity does not weaken the justification for a tax on animal products obtained with the use of antibiotics: the social cost of antibiotic use is the same regardless of the purpose for which antibiotics are used, and in any case consumers are aware of the cost they are imposing on society and are in the position to avoid contributing to such cost. However, we grant that, when needed for genuinely therapeutic purposes, the reasons for using antibiotics are stronger than in other cases; such reasons can be taken to counteract, at least to a certain extent, the deontological reasons for requiring individuals to internalize the cost of antibiotic use. Thus, we suggest that in the case of meat produced with *therapeutic* use of antibiotics the tax could be less heavy than in the case of prophylactic or growth promoting use of antibiotics.

It is worth pointing out, however, that even therapeutic use often involves the administration of antibiotics to healthy animals, and therefore the distinction between necessary and unnecessary use of antibiotics for health purposes is blurred. In particular, in commercial, large scale production systems, treatment is typically applied on a population level, and not on an individual level: when a farmer finds, for instance, one meat-chicken showing symptoms of a respiratory disease, even in the most strictly regulated settings, the vet will prescribe to treat the whole flock of, say, 40.000 chickens.

Besides, the costs of consumption of non antibiotic-free meat are not confined to antibiotic resistance. Other types of costs need to be factored in. For example, large scale livestock production systems threaten rural livelihood (through the displacement of small scale farms that decreases employment numbers in agriculture) and local food security in developing countries (Garces 2002). Livestock production is also a major source of ammonia/CO2, methane and other environmental pollutants. These are all externalities of meat production that, according to the deontological arguments presented here, consumers might legitimately be requested to (at least partially) internalise, and for which they might legitimately be requested to compensate society.

### Cost-Benefit Analysis

Arguments for a tax on animal products obtained with the use of antibiotics that are based on a cost-benefit analysis are more straightforward than the deontological ones in that they do not require appealing to, and therefore arguing for, consumers’ moral responsibility for the acceleration of antibiotic resistance. From the perspective of a cost-benefit analysis, a tax would be justified simply if the overall benefits that would result from it outweighed the costs.

Now, there are different types of costs that a tax on non antibiotic-free meat would create. A type of cost consists in the fact that people would either have to pay more for meat or consume less of it (we assume for the sake of argument that consuming meat confers at least some benefit on the consumer) or both. This state of affairs would probably also entail an economic cost for producers, if consumption of meat dropped significantly as a consequence of the increase in the costs of meat. However, the benefits that this state of affairs would bring about, in terms of reduced social and individual harms, are potentially significant. Antibiotic resistance represents a high cost not only for people currently living, but also for future generations, who might not avail themselves of antibiotics to treat serious infections, as the examples provided in the introductory section suggest. Considering both present and future generations, the number of people harmed by antibiotic resistance is potentially enormous. By contrast, it is not clear that the costs of reduced meat consumption and production would be comparably large. It is therefore reasonable to claim that the benefits of a tax would outweigh the costs if the tax resulted in a significant reduction of antibiotic use in animal farming and therefore in a significant deceleration of antibiotic resistance (which does not necessarily mean a reduction in sales of meat, if alternatives to antibiotic use are adequately supported in a way that would make their prices competitive: we will return to this point below). We argue that a tax could have this desirable effect. More in particular, there are two types of benefits that could derive from a tax on non-antibiotic free meat, namely: (a) the likely effects of the tax on consumers’ behaviour, in terms of reduced consumption of meat produced with the use of antibiotics, and (b) the fact that revenue generated through the tax could be used to fund alternatives to antibiotics: supporting such alternatives, besides yielding a benefit in terms of deceleration of antibiotic resistance, would minimize the economic costs for farmers of transitioning to alternative methods of animal husbandry. Let us consider these two types of benefits more in details.

As for the former type, it is likely that the tax would discourage people from consuming animal products obtained with the use of antibiotics, which would in turn contribute to the deceleration of antibiotic resistance. Without such tax, the price of antibiotic-free meat would probably be higher than the price of meat produced with the use of antibiotics, because, as we shall see below, alternatives such as vaccines are often more expensive than antibiotics. Thus, without the tax, many consumers would likely be oriented towards consuming meat produced with the use of antibiotics in consideration of its lower price. Although we saw above that a significant proportion of consumers would be willing to pay more for antibiotic-free meat, it is still the case that more consumers would opt for antibiotic-free meat if antibiotic-free meat cost the same or less than meat produced with use of antibiotics. Thus, reducing or, ideally, eliminating the difference in price between the two types of meat through taxation would significantly reduce the consumption of meat produced with the use of antibiotics and therefore reduce consumers’ contribution to antibiotic resistance.

Of course, other things being equal, there would still be an overall increase in average price of animal products, since consumers would have to either pay the tax, or purchase the more expensive antibiotic-free meat. Some might think this is a morally significant type of cost of the tax, for example because it makes it more difficult for individuals with low income to be adequately nourished or to enjoy the pleasures of meat-eating. There are two responses to this objection. First, as we suggest below, use of alternatives to antibiotics could be financially supported through the revenue generated through the tax: it might thus be possible to reduce the cost of producing antibiotic-free meat to such an extent that its price on the market would not only be lower than the price of the taxed meat, but perhaps even lower than the current price of meat obtained with the use of antibiotics. Admittedly, one might reply here that it is unlikely that the revenue generated through the tax would be so large as to allow to incentivize alternatives to antibiotics to such an extent that antibiotic-free meat would be priced less than the meat currently on the market. However—and this is the second response to the objection—even if meat will end up being overall more expensive as a consequence of the introduction of the tax, to the extent that the tax would have costs for (prospective) meat eaters, it is doubtful that these individuals would have legitimate grounds for complaint. After all, in the absence of the tax and therefore of incentives to consume antibiotic-free meat, there would be increased costs for others in the form of increased antibiotic resistance. As the deontological arguments discussed above suggest, it seems preferable that the costs of meat eating are borne by those who choose to engage in this practice than that they are borne by others.

The second type of benefit of a Pigovian tax on consumption of animal products obtained with the use of antibiotics would occur if, as we suggest in “Tax to Fund Alternatives to Antibiotics Used for Prophylactic or Therapeutic Purposes” section and “Tax to Fund Alternatives to Growth Promoting Antibiotics” section below, the revenue generated through such tax were used to fund alternative methods of animal husbandry that would replace antibiotic use. If effective alternatives to antibiotics were introduced, meat production would no longer involve a cost in terms of antibiotic resistance, and this in turn would eliminate the harm to society and to others of consuming meat; besides, if such alternatives were adequately subsidized, the cost to producers of transitioning to a system in which alternatives replace antibiotic use would be significantly reduced. Thus, the cost-benefit analysis of using the tax to fund alternatives to antibiotic use in animal farming seems to be favourable.

Along similar lines, and considering the cost in terms of antibiotic resistance of antibiotic consumption, some have argued that antibiotics sold for use in humans should be taxed (Littmann and Viens 2015; Anomaly 2013; Herrmann and Laxminarayan 2010), and that revenue from the tax could be used to fund research on new, effective antimicrobials (O’Neill 2016, pp. 12–14). In this way, antibiotic consumers would be financially supporting measures aimed at the preservation of antimicrobial effectiveness, thus contributing to offsetting the cost of the antibiotic resistance they are contributing to create. The same types of reasons that support taxing antibiotics in humans—namely the need to reduce or ideally eliminate the negative externality of current antibiotic use—would support taxing meat produced with the use of antibiotics. Granted, the measure through which the negative externality is reduced would obviously be different in the two cases. In the case of human consumption, where effective antibiotics are necessary to treat infections, the aim is to replace current antibiotics with new and more effective antibiotics; in the case of animal consumption, the aim is to replace current antibiotics with alternative methods of animal husbandry which, as we shall see in the next sections, would significantly reduce the need for prophylactic, therapeutic, or growth promoting use of antibiotics.

In the next two sections we will explain in more detail how the revenue generated through taxation could be used to fund alternative methods of animal husbandry that do not require antibiotic use. Granted, there might be other good uses of the revenue generated through the tax, perhaps uses which have nothing to do with funding alternatives to antibiotics. But using the revenue to fund alternatives to antibiotic use would strengthen the justification for such a tax, making it more likely to be accepted by consumers and population at large and therefore more politically palatable.

## Tax to Fund Alternatives to Antibiotics Used for Prophylactic or Therapeutic Purposes

In some countries antibiotics are currently used, officially, only for prophylactic or therapeutic purposes. This is the case, for example, in the EU countries, where GPAs have been banned since 2006. However, even in these countries the use of antibiotics continues to be significant (O’Neill 2015, p. 17) In order to reduce non growth-promoting use of antibiotics while maintaining a flourishing market in animal products it is necessary to implement effective alternatives to prophylactic and therapeutic uses of antibiotics. Such alternatives would allow cutting antibiotic use in animal farming without significantly increasing infectious disease rates in animals. Fortunately, alternatives to prophylactic and therapeutic use of antibiotics in animal farming are available. We provide here some examples of possible alternatives to antibiotic use for prophylactic or therapeutic purposes. We then suggest that revenue from a tax on meat produced with the use of antibiotics could be used to fund such alternatives.

One alternative is the use of vaccines to prevent the infections that would otherwise have to be prevented or treated with antibiotics. According to the WHO, “sustainable husbandry practices, including the use of vaccines, can reduce infection rates and dependence on antibiotics, as well as the risk that antibiotic resistant organisms will develop and spread through the food chain” (WHO 2015, p. 10).

Regulation of stocking density could drastically reduce the need to use AB. Barn hygiene status, including air quality, the moisture of litter substrate, and pathogen load are factors that are very closely linked to stocking density. (Besides, keeping animals on slatted floor or wire floor gives rise to very serious animal welfare issues, especially because animals are no longer able to perform species-specific behaviour).

Another strategy to prevent infections is that of using nutritional supplements instead of antibiotics: for example, zinc oxide is commonly used in pigs (Wierup 2001), probiotics, dicarboxylic acids, enzymes and plant-derived products including saponins, tannins and essential oils in ruminants (Jouany and Morgavi 2007) and prebiotics, probiotics (Patterson and Burkholder 2003), herbal additives (Sarica et al. 2005) and others are either used, or studied as potential alternatives to antibiotics use, in meat chickens.

A fourth alternative strategy is that of breeding animals with better immunity to common diseases, which would reduce the need for therapeutic use of antibiotics. There is a large body of literature on attempts to breed for disease resistance in various species (Axford and Owen 1991; Stear et al. 2001; Shook 1989). Especially in extensive systems (e.g. outdoor systems for laying hens, broilers, pigs or aquaculture such as salmon rearing in lake or ocean pens) where contact to pathogens from the outside cannot be avoided altogether (it can never be avoided completely unless nano-filters are installed in barn vents), the use of better adapted breeds carries various potential advantages over the administration of antibiotics. Advantages include lower mortality rates, improved animal health and welfare, and lower costs in production, as the costs of purchasing and administering antibiotics (vet bills if prescribed and labour costs) would be decreased.

As is easy to imagine, however, most of these alternatives, as well as the transition to a system that employs these alternatives, do have a cost, and often a higher cost for producers than simply using antibiotics. For example, vaccines are currently very expensive compared to antibiotics as a prophylactic measure (O’Neill 2015, p. 34). Thus, we suggest that revenue from a consumption tax on animal products obtained with the use of antibiotics *could* be used to subsidize the transition to a system of meat production characterized by better infection control, better hygiene, reduced stock density, nutritional supplement instead of antibiotics, breeding of animals with better immunity to common diseases, and a wider use of vaccines. In other words, revenue from the consumption tax could be used to fund the transition to a system in which the need for prophylactic or therapeutic use of antibiotics is strongly reduced. The cost-benefit analysis we have discussed in the previous section does support this type of use of the revenue generated through the consumption tax, and, as said above, using the revenue to fund alternatives to antibiotic use would make the tax more likely to be accepted and more palatable. In this way, the negative externality represented by antibiotic resistance would in the long run be reduced as antibiotics are replaced by alternative methods.

The final aim of this type of consumption tax is the transition to a system in which the source of the tax revenue—namely the use of antibiotics in animal husbandry—is progressively eliminated. In order to favour a complete transition, then, other sources of subsidization are necessary. In particular, states should subsidize alternative methods of animal husbandry that do not require the use of antibiotics, independently of the revenue generated by the proposed tax.

## Tax to Fund Alternatives to Growth Promoting Antibiotics

Part of the reason why antibiotic consumption remained high in EU after the 2006 ban on GPAs was the lack of clarity and of transparency regarding what represents therapeutic and non-therapeutic use (O’Neill 2015, p. 17). Thus, for example, after the EU ban and before the Dutch Government mandated a 50% reduction on antibiotic use in animals in 2009, the “therapeutic” use of antibiotics increased in the Netherlands to such an extent that the total use of antibiotics remained static despite the EU ban (Cogliani et al. 2011, pp. 276–77). Denmark and the Netherlands eventually managed to reduce the use of antibiotics to 50 mg per kg of livestock while maintaining a flourishing market of meat production and export (Denmark is currently one of the largest pork exporters in the world). However, the use of antibiotics in other EU countries has remained nearly three times as high—being today around 146.7 mg/kg (O’Neill 2015, p. 20). It is not unreasonable to suppose that much of such use is for non-therapeutic purposes, and perhaps not only for prophylactic, but also for growth promoting purposes.

Whatever explains the high antibiotic use in animal farming in the EU, we know for sure that antibiotics are currently used for growth promoting purposes in the US and in many other countries. Interestingly, the use of GPAs in animals remains high in spite of the emerging evidence suggesting that “antibiotics used as growth promoters do not have as much economic benefit as previously thought, particularly in countries with advanced farming techniques” (O’Neill 2015, p. 15). For example, a study has shown that in the US the use of antibiotics in livestock for production purposes is associated with only a 1–3% increase in productivity, which is considered statistically insignificant; besides, it has been estimated that a restriction on GPA use would reduce production of only 1–2% and generate a decline in the value of production of less than 1% (Sneeringen et al. 2015). Therefore, a restriction on antibiotic use for production purposes would probably not have devastating effects on the economy. Actually, Graham and colleagues have found “no basis for the claim that the use of GPAs lowers the cost of production” in the case of commercial broiler chickens in the US. Rather, given the cost of antibiotics and the very limited positive production changes associated with GPAs, they conclude that “use of GPAs in poultry production is associated with economic losses to the producers”, which they quantify in 0.0093$ per chicken (Graham et al. 2007, p. 86). Thus, according to Graham and colleagues, “[c]ontrary to the long-held belief that a ban against GPAs would raise costs to producers and consumers, these results using a large-scale industry study demonstrate the opposite. GPA-associated gains in feed conversion ratios were insufficient to offset the cost of the biological agents” (Graham et al. 2007, p. 86). In fact, agricultural data from Sweden indicated no loss in production of meat after the country banned GPAs in 1986 (Cogliani et al. 2011, p. 275). Other studies have shown that in the case of meat birds (broiler chickens) “under good production conditions it is possible to reach good and competitive production results for the rearing of poultry without the continuous use of antibiotics in feeds” (Castanon 2007).

It is true that previous studies had found increases in body weight of poultry up to 11% (with penicillin) (e.g. Dafwang et al. 1984); however, many of these studies were conducted in the 1980s or earlier, i.e. before the introduction of innovations in poultry science which favoured growth without the use of antibiotics, such as selective breeding and changes in animal husbandry and feed formulas (Graham et al. 2007, p. 81). It is therefore plausible to assume that reduction or even elimination of GPAs *where advanced breeding and husbandry techniques are in use* would not have significant economic consequences for meat producers.

However, the economic consequences of eliminating GPAs would probably be greater in other contexts, where advanced techniques are less accessible and therefore less used. Studies have shown that while the growth response to GPAs is reduced in optimized production systems (Laxminarayan et al. 2015, p. 14; O’Neill 2015, p. 15), i.e. in systems with good housing and hygiene and optimal nutrition and health (Laxminarayan et al. 2015, p. 14; Graham et al. 2007, p. 80), the response is significant in systems with less developed hygiene and production practices and with lower level of animal well-being (Laxminarayan et al. 2015, p. 14, p. 35; Hill et al. 1953; Coates et al. 1951). Even within the same country, such as in the US, it has been estimated that a ban on GPAs “would impact producers differentially, according to location, farm size, contracting arrangements, production practices, etc.” (Laxminarayan et al. 2015, p. 35). Accordingly, we suggest that revenue from consumption taxes could be used to subsidize the development of better hygiene and production practices where this is most needed, so as to make GPAs less effective and therefore less appealing to producers and farmers who have so far adopted less advanced husbandry techniques.

It is worth noting that the factors that account for an optimized production system are typically less developed in lower income countries (Laxminarayan et al. 2015, p. 5). GPAs are therefore more effective and more appealing in those countries. Therefore, assuming the revenue generated through a consumption tax on animal products obtained with the use of antibiotics would be so large as to allow this, such revenue could ideally be used to at least partially subsidize the development of better production strategies also in these countries, and not just in the countries where animal products are purchased. After all, antibiotic resistant bacteria know no national boundaries. In today’s globalized world, resistant bacteria can easily spread globally because of the current rates of international travels and of commercial traffic between countries. It is therefore in everybody’s interest that antibiotic resistance be contained to the greatest extent possible in any part of the world. Thus, there seem to be good reasons for using part of the revenue from a tax on consumption of animal products obtained with the use of antibiotics to fund the transition to a system where the negative externality of antibiotic resistance is significantly reduced, i.e. to reduce farmers’ and producers’ dependence on GPAs where such dependence is greater.

We acknowledge, however, that the revenue generated through the tax would probably not be so large as to allow to fully (or perhaps even significantly) fund the transition to a production system characterized by reduced dependence on GPAs, as well as by reduced dependence on antibiotics used for prophylactic or therapeutic purposes. Therefore, independent forms of subsidization could (and should) be provided by states, including subsidization of alternatives to GPAs in poor countries. Our claim is simply that revenue from the tax could at least *contribute* towards reducing dependence on antibiotics in animal farming.

## Collective and Individual Responsibilities

In this section we are going to address a possible objection to our arguments in favour of a tax on animal products obtained with the use of antibiotics. The objection is that each individual contribution to antibiotic resistance through individual meat consumption is imperceptible; accordingly, individuals are not individually causally responsible for antibiotic resistance. Because her contribution makes only an imperceptible difference, i.e. it is causally irrelevant,—so the objection might go—an individual does not have an individual moral obligation to abstain from consuming meat produced with the use of antibiotics. Single individuals should therefore not be required to compensate society for a harm for which they cannot be held morally and causally responsible. In contrast, we argue that the tax would be fair despite the imperceptibility of individual contributions to antibiotic resistance through individual consumption of meat.

Let us start by acknowledging the fact that people who buy meat produced with the use of antibiotics are *collectively*, and not individually, responsible for antibiotic resistance caused by such use: only when considered collectively do their purchases support the practice of feeding antibiotics to animals. Any single purchase would probably not make a relevant difference to the total amount of antibiotics used in animal farming and therefore to antibiotic resistance. Broadly speaking, to be responsible for a certain negative outcome, such as a negative externality, means, in the retrospective sense of responsibility, to be blameworthy for the outcome and, in the prospective sense of responsibility, to have a moral obligation to either prevent that outcome or to make up for it. As we have seen above, in the case of consumption of meat produced with the use of antibiotics, “making up” means compensating those affected by the negative externality of antibiotic use. When there is an obligation to make up for a certain negative outcome, retrospective and prospective responsibilities are logically and morally connected, in the following sense: from a logical point of view, the moral (prospective) responsibility to “make up” for a certain outcome presupposes the retrospective responsibility for that outcome (otherwise it would not be a case of “making up”); and, from a moral point of view, being blameworthy for something generates a prima facie moral reason to make up for it. The deontological argument for a tax on antibiotic resistance we have presented above is based on this moral claim. However, we can see now how that deontological argument, *by itself*, only establishes collective duties, and not individual duties. Let’s see in more details why.

As said above, the fact that the responsibility in question is collective means that any single purchase of meat is unlikely to make a significant difference to the amount of antibiotics used in animal farming, and therefore to antibiotic resistance. It is often held that “ought” implies “can”: someone cannot have the obligation to bring about or to prevent a certain outcome if she does not have the causal power to realise or to prevent it. And in fact it has been argued that in similar cases, i.e. when one’s contribution to a negative externality is imperceptible and therefore does not have any causal significance (such as for example in the case of one’s contribution to air pollution through driving on Sundays just for fun), such contribution also lacks moral significance, and therefore one does not have a moral obligation to refrain from contributing to the externality—although states might have the right or the duty to intervene to prevent or discourage individual behaviours that contribute to the externality (Sinnott-Armstrong 2005). Lack of individual causal power with regard to the collective effect explains why the deontological argument presented above, by itself, only establishes collective, but not individual moral duties. Therefore, it might be added, since one cannot be blameworthy for a negative externality in the case of imperceptible individual contributions, one does not have the related prospective moral obligation to contribute to the internalization of the negative externality or to compensate society for one’s contribution to a negative externality—although the state might have an independent right or duty to force people to internalize the externality or to compensate society, for example on the basis of considerations about the likely benefits of such policies. Our problem is exactly that of determining whether, in the case of antibiotic resistance caused by human consumption of meat, such interventions by the state would not only be justified on the basis of the benefits they might yield, but would also reflect a moral obligation to make up for the negative externality that each individual has, regardless of the actual impact on antibiotic resistance of her choice to consume meat produced with the use of antibiotic. If individuals had this moral obligation in spite of the imperceptibility of their contribution to the negative externality—i.e. if the deontological argument applied to individuals, and not just to collectives—then the justification for taxation would be stronger than it would be if it were only based on considerations about the benefits of such policy.

Now, the view that individuals are not blameworthy for a negative externality caused by a collective and to which they make an imperceptible contribution can be challenged. Accordingly, we can also challenge the view that such individuals do not have the related prospective moral obligation to contribute to the internalization of a collective negative externality or to compensate society for it.

Let us first examine one possible argument against such views which, although appealing, seems to turn on an empirical issue that we are happy to leave open, and which therefore we will not take as a conclusive argument to the effect that an individual is blameworthy for a negative externality caused by a collective (those interested only in the argument we propose can skip the rest of this paragraph and jump directly to the next). Following Shelly Kagan’s argument against purchasing meat (Kagan 2011), one might say that there is a chance, although a very small one, that each individual act of meat consumption does make a difference to the number of animals raised and killed in animal farming, and therefore to the amount of antibiotics used in animal farming. This is because, as Kagan has argued, any purchase of meat has a small chance of being the one that triggers a new order of meat by the butcher, and any new order of meat causes more animals to be brought into existence and raised in factory farming. Accordingly, Kagan has argued that the expected utility of each purchase of meat remains negative, although the chances that the negative effect occurs with any purchase are very small. Kagan was referring not to the negative utility of antibiotic resistance, but to the negative utility of inhumanely raising and killing animals for food production. However, one might apply the same argument to the case of antibiotic use in animal farming and say that any purchase of meat might be the one that causes more animals to be brought into existence, and that more animals brought into existence and raised in contemporary animal farming also cause more antibiotics to be used. Accordingly, one might continue, also the expected utility, in terms of contribution to antibiotic resistance, of any purchase of meat is negative. From a consequentialist perspective, then, consuming animal products obtained with the use of antibiotics might still be impermissible. One might object to this consequentialist argument, however, by pointing out that, contrary to the case of the negative utility of animal suffering, any additional order of meat would not make any significant difference to the amount of antibiotics used in animal farming and therefore to the acceleration of antibiotic resistance: one might say that the additional number of animals that would be brought into existence and raised in factory farming as a consequence of any new order of meat by a butcher would still be too small to make a *relevant* difference to the amount of antibiotics used in factory farming, i.e. a difference that would have significant consequences in terms of acceleration of antibiotic resistance. Whether this objection works is largely an empirical issue: one could reply that, as a matter of fact, any small increase in the number of animals brought into existence and raised in contemporary factory farming does increase the amount of antibiotic used in animal farming to an extent that it does have significant consequences in terms of antibiotic resistance. As said above, this is an empirical issue which we are happy to leave open. For those who are sceptical about the application of Kagan’s argument to the case of antibiotic use, we provide here a different, and non-consequentialist, argument for why individuals might still be considered individually blameworthy for their contribution to antibiotic resistance through consumption of meat, even assuming that their contribution to antibiotic use is imperceptible. The argument is the following.

When a collective is responsible for a negative outcome (or a negative externality) produced through the *aggregate* actions of individuals, the collective is to be identified not as an independent “super-entity”—as might be the case when we talk, for example, of a state or of a corporation. Such “super-entities” are characterized by internal constitutions or codified decision procedures that, at least on some views, qualify them as moral agents, and in virtue of which they might be attributed responsibility independently of attribution of responsibility to their members (e.g. List and Pettit 2011; Pettit 2007). The type of collective we are interested in is simply a random aggregation of individuals, each of whom independently contributes, however imperceptibly, to the negative collective effect. Thus, attributing moral responsibility to random aggregations of individuals cannot but mean attributing moral responsibility to their individual members, because there is no other entity that can be considered a legitimate bearer of moral responsibility—in other words, collective responsibility of random collections is distributive, at least, as Virginia Held put it, when it is obvious to a reasonable person what individuals ought to have done to prevent the undesirable outcome (Held 1970). The problem is how to justify such attribution of moral responsibility to individuals, considering that each individual contribution makes only an imperceptible difference to the collective effect for which the collective is held responsible. Why, and to what extent, would each individual be blameworthy for her contribution to antibiotic resistance through consumption of animal products, if such contribution only makes an imperceptible difference to the development of antibiotic resistance? And does any individual have a moral obligation to contribute to internalizing the externality or to compensate society for it, if her contribution to the production of that externality is negligible?

We can answer such questions by shifting our attention from consequentialist considerations about the actual impact each individual has on the collective effect to considerations of fairness, and more precisely of fairness in the distribution of the burdens entailed by collective responsibility. Fairness is an important value that, in general, we do and should promote, and which should therefore play a role also in defining the relationship between collective and individual responsibility. Thus, it is reasonable to assume that individual members of a collective that is morally responsible for a negative outcome ought to take on themselves a *fair* share of the burdens entailed by the collective responsibility, both in a retrospective sense—i.e. they should be considered partially blameworthy for their contributory behaviour, even if their impact is imperceptible-, and in a prospective sense—i.e. they should do their fair share to prevent the outcome, or to make up for it. Accordingly, we can say that individuals are not responsible for the actual impact of their contributions to antibiotic resistance, but they are responsible for the act itself of contributing. Such an act is rendered morally relevant by the fact that it violates the requirement to make one’s *fair* contribution to fulfilling the collective obligation to prevent antibiotic resistance, rather than by consequentialist considerations about its actual impact on the collective effect.

Steven Sverdlik introduced a useful conceptual distinction between responsibility for outcomes and responsibility for actions: as he argued, while collectives can be held responsible for collective *outcomes*, individual members of the collective, even if they do not have the causal power to affect such outcomes, can be held responsible for their contributory *actions* (Sverdlik 1987). Our argument about the moral obligation to do one’s fair share to fulfil a collective obligation, or to take on oneself a fair share of the burdens entailed by a collective obligation, points in the same direction. The collective of people consuming meat produced with the use of antibiotics might be held, as a collective, morally responsible for antibiotic resistance in the sense that all individuals are, individually, morally responsible for the contributory actions of consuming meat. Such actions become morally relevant, and therefore render agents proper subjects of moral responsibility (i.e. blameworthiness), because they violate the fairness-based demand to do one’s fair share to fulfil a collective obligation—namely, in this case, the collective obligation not to accelerate antibiotic resistance.

Distributing fairly the burdens of the collective responsibility of aggregative collectives is consistent with what Gerald Gaus called the “Public Harm Principle”, according to which

If (1) an accumulation of X-ing sets back other people’s welfare interests, and if (2) the harm is serious enough such that its prevention warrants limiting the liberty to X (either by regulating or prohibiting X-ing), then (3) everyone should carry their fair share of the burden (…), and (4) everyone who Xs is responsible for a share of the harm done (Gaus 1999, p. 197)

Thus, any individual who purchases animal products obtained with the use of antibiotics is morally responsible for failing to do her fair share to avoid the negative externality of antibiotic resistance: the imperceptibility of her contribution to the collective effect does not mean that she is not blameworthy. But if an individual is blameworthy for her contribution to a negative collective externality, then, in virtue of the moral connection between retrospective and prospective moral responsibility, she has a moral obligation to make up for her contribution. Accordingly, a tax could be seen as a way of ensuring that each individual who, in violation of the demands of fairness, consumes meat produced with the use of antibiotics fulfils her moral obligation to contribute to compensating society for the externalities associated with antibiotic use.

## Conclusions

Use and abuse of antibiotics in animal farming is one of the main causes of the acceleration of antibiotic resistance. Once informed about the consequences of antibiotic use in terms of antibiotic resistance, individuals who choose to consume animal products which they know have been produced with the use of antibiotics are (1) *collectively* morally responsible (i.e. blameworthy) for the acceleration of antibiotic resistance caused by antibiotic use in animal farming, but also (2) *individually* morally responsible for their contribution to such outcome, even if each individual act of consuming animal products makes a very small, imperceptible, difference to the development antibiotic resistance. Such individual responsibility implies that each individual has a duty to “make up” for the contribution for which she is morally responsible, i.e. to contribute to the internalization of the negative externality of antibiotic resistance and to compensating society for the harm that such negative externality creates. While these represent deontological considerations in favour of a tax on consumption of meat produced with the use of antibiotics, there are also considerations based on a cost-benefit analysis in support of such a tax: the benefits of the tax could outweigh the costs because (a) the tax would discourage people from consuming meat produced with the use of antibiotics, thus contributing to slowing down antibiotic resistance; and (b) because it would generate revenue that could be used to support alternatives to the use of antibiotics in animal husbandry for prophylactic, therapeutic, and growth promoting purposes, thus reducing the economic cost for producers of transitioning to an antibiotic-free production system and, consequently, contributing to reducing antibiotic resistance caused by use of antibiotics in animal farming. Thus, there are both deontological arguments and arguments based on a cost-benefit analysis for taxing animal products obtained with the use of antibiotics.

## Footnotes

1 It might be argued that if the tax were used in this way, it would not qualify as a form of compensation, since the money would not be transferred to those who are harmed by antibiotic resistance. Those individuals would still suffer uncompensated harms. We agree; however we believe it nevertheless makes sense to think of the tax as a form of compensation since *society at large* is compensated for the costs that it bears due to anti-biotic resistance.

2 Although adding additional information can also have costs—for example, information overload may lead to confusion or to less attention being given to information—and at some point, the costs of adding additional information may outweigh the benefits.
